# Bias polarity dependent low-frequency noise in ultra-thin AlO_x_-based magnetic tunnel junctions

**DOI:** 10.1038/s41598-024-59675-3

**Published:** 2024-06-13

**Authors:** Chun-Yen Chen, César Gonzalez-Ruano, Isidoro Martinez, Farkhad G. Aliev, Dah-Chin Ling, Yu-Hui Tang, Jhen-Yong Hong

**Affiliations:** 1 Department of Physics, National Central University, Taoyuan City, 320317 Taiwan; 2 Departamento Física Materia Condensada, C03, INC-Instituto Nicolas Cabrera and IFIMAC-Condensed Matter Physics Center, Universidad Autónoma de Madrid, Madrid, 28049 Spain; 3 Departamento FAIAN, E.T.S.I. Aeronáutica y del Espacio, Universidad Politécnica de Madrid, Madrid, 28040 Spain; 4 Department of Physics, Tamkang University, Tamsui Dist., New Taipei City, 251301 Taiwan

**Keywords:** Electronic and spintronic devices, Spintronics

## Abstract

We exploit bias polarity dependent low-frequency noise (LFN) spectroscopy to investigate charge transport dynamics in ultra-thin AlO_x_-based magnetic tunnel junctions (MTJs) with bipolar resistive switching (RS). By measuring the noise characteristics across the entire bias voltage range of bipolar RS, we find that the voltage noise level exhibits an bias polarity dependence. This distinct feature is intimately correlated with reconfiguring of the inherently existing oxygen vacancies (${V}_{O}^{..}$) in as-grown MTJ devices during the SET and RESET switching processes. In addition, we observe two-level random telegraph noise (RTN) with a longer and shorter tunneling length in the high resistance state (HRS) and low resistance state (LRS) at a low bias voltage. The intrinsic voltage fluctuations of RTN arise from the dynamics of electron trapping/de-trapping processes at the ${V}_{O}^{..}$-related trap sites. Notably, the RTN magnitude is similar in LRS but nonidentical in that of HRS for different bias polarity. These findings strongly suggest that the inherent ${V}_{O}^{..}$ are distributed near the top CoFe/AlO_x_ interface in the HRS; in contrast, they are expanded to the middle region of the AlO_x_ in the LRS. More importantly, we demonstrate that the location and distribution of the inherent ${V}_{O}^{..}$ can be electrically tuned, which plays an essential role in the charge transport dynamics in the ultra-thin AlO_x_-based MTJs and have significant implications for developing emergent memory and logic devices.

## Introduction

Bipolar RS has been reported in various transition-metal oxides^1^, including hafnium oxide (HfO_2_)^2^ , titanium dioxide (TiO_2_)^3^, nickel oxide (NiO)^4^, and aluminum oxide (Al_2_O_3_)^5^. The electric field-induced migration of oxygen ions (O^2−^) or vacancies (${V}_{O}^{..}$) is recognized as the main mechanism responsible for the RS in these materials^6^. However, some studies on oxide-based RS devices have suggested that the electron injection/extraction^7^ and the charge trapping/de-trapping with the ferroelectric-like behavior^8,9^, rather than field-driven ionic motions, may take place in an ultra-thin amorphous AlO_x_. In addition, other reports have shown that the O^2−^ could migrate by the annealing effect to create ${V}_{O}^{..}$^10,11^. Nevertheless, our previous studies have demonstrated the coexistence of the RS along with the tunneling magnetoresistance (TMR) effect in AlO_x_-based MTJs, suggesting that both the resistance states and TMR ratio could be tuned by applying a bias voltage^12^. Therefore, further investigations to elucidate the underlying mechanism of RS in AlO_x_-based devices are of great practical importance.

LFN spectroscopy is a highly sensitive and non-invasive technique that can identify the nature of charge carriers, the presence of traps, and the role of the interfaces. It has been widely employed to probe the transport dynamics of MTJs with RS^13,14^. Our previous work reported that the 1/*f* noise level change in the AlO_x_-based MTJ devices could be attributed to electron trapping/de-trapping processes in an ultra-thin (~ 1.5 nm) AlO_x_^15^. Despite extensive investigations on RTN caused by defect-mediated charge trapping and de-trapping processes neighboring the conductive filament (CF) in RS systems with thick oxide layers^16^, the conduction mechanism of charge carriers through a tunneling barrier with the existence of intrinsic trap states associated with ${V}_{O}^{..}$ in an ultra-thin AlO_x_ layer remains elusive. Hence, the exploration of the LFN across the entire bias voltage range, along with low bias RTN, of bipolar RS in ultra-thin AlO_x_-based MTJ devices can pave a way toward a better understanding of the charge transport dynamics in the devices and, more importantly, optimizing device performance, reliability, and stability.

In this work, we investigate the bias voltage dependence of LFN in the MTJ studied. We find that the voltage noise power spectral density (PSD) level is with respect to the bias polarity in the resistive switching between HRS and LRS. Notably, we observe bias polarity dependent RTN fluctuations arising from the capture and emission of carriers at localized traps, with two-level time series and large/short cut-off frequency in the HRS/LRS at a low bias voltage, respectively. We identify that the electrically tunable location and distribution of the inherent ${V}_{O}^{..}$ s are essential ingredients for observing the results mentioned above and the main transport mechanism in the ultra-thin AlO_x_-based MTJs.

## Sample fabrication and experimental details

**Figure 1:**
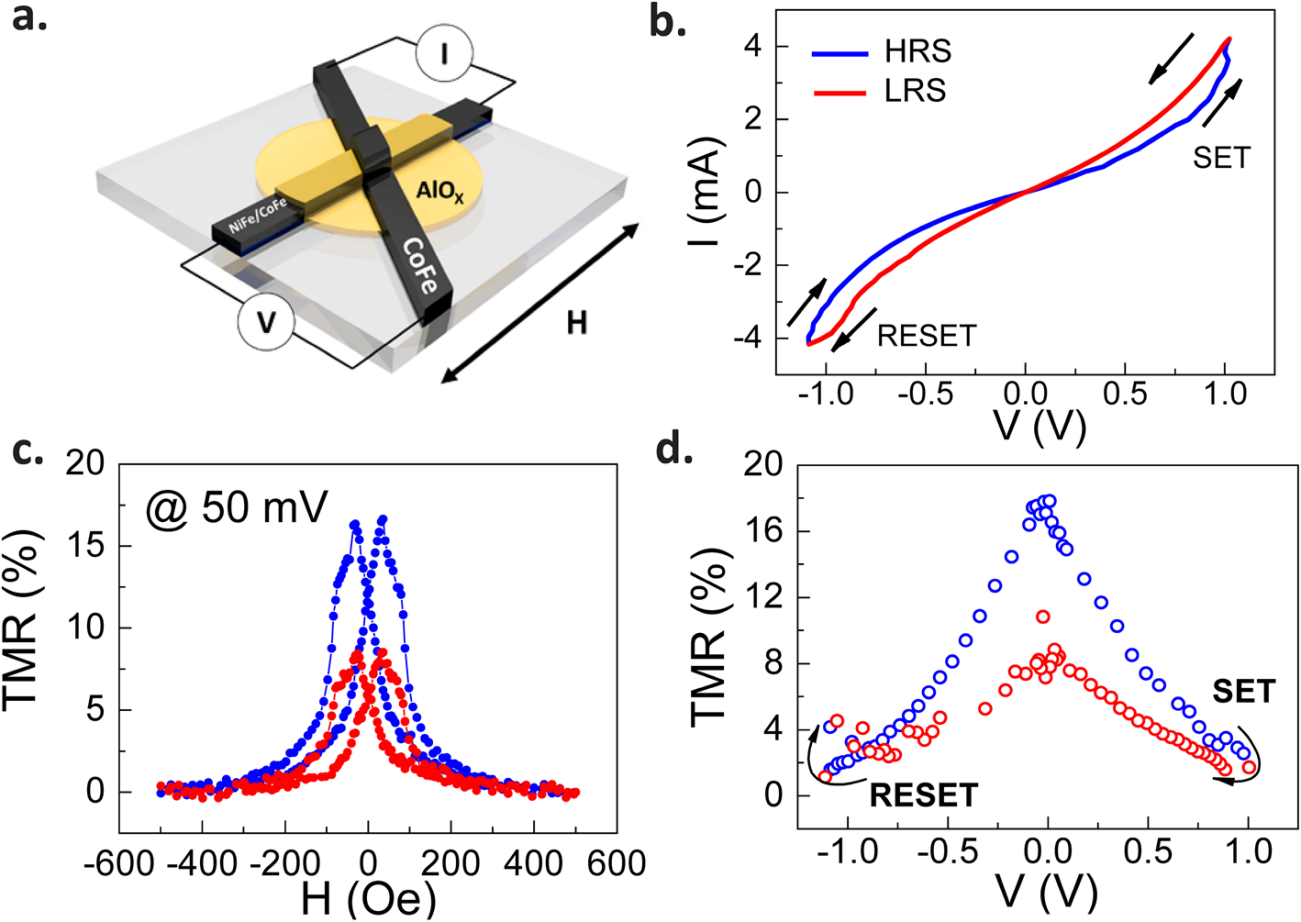
(**a**) The diagram of NiFe/CoFe/AlO_x_/CoFe MTJ structure (**b**) The I–V curve with bipolar resistive states: HRS (blue line) and LRS (red line). The arrows indicate the voltage sweeping direction of the SET and RESET processes. (**c**) The TMR ratio versus magnetic field taken at 50 mV in the HRS and LRS. (**d**) The voltage dependence of the TMR ratio measured along with the bipolar switching I-V curve shown in (**a**). The data was extracted from the peak value of the TMR ratio-vs-*H* plot at different bias voltages.

The MTJ studied with layer sequence of NiFe (10 nm)/CoFe (15 nm)/AlO_x_ (1.5 nm)/CoFe (30 nm) was deposited on a glass substrate and patterned in a crossbar configuration with the junction area of 150 μm × 150 μm in an ultra-high vacuum (UHV) sputtering chamber with a base pressure of 10^–8^ torr, as shown in Fig. 1a. The ferromagnetic (FM) layers were deposited in a working Ar pressure of 5 × 10^–3^ Torr. The thin AlO_x_ layer was fabricated by partially oxidizing the aluminum layer under an oxygen atmosphere at a pressure of 0.2 Torr for 120 s, followed by an oxygen plasma treatment at an oxygen pressure of 0.03 Torr for 60 s. This two-step oxidation process makes oxygen vacancies inherently exist in the thin AlO_x_ layer. The top CoFe layer serves as the hard FM layer, and the bottom NiFe/CoFe layer acts as the soft FM layer due to a weaker coercivity of NiFe. For the magneto-transport measurements, the junction voltage was acquired by a data acquisition (DAQ) board with the bias source supplied by Keithley 2400 source measure unit (SMU). The noise signal was split into two channels for the cross-correlation method in the low-frequency noise measurements. These signals were amplified by a two-stage amplification scheme through homemade pre-amplifiers and SR560 commercial voltage amplifiers. The time series and the analyzed spectrum were measured by an SR780 spectral analyzer. Note that our system's noise floor was calibrated at the level of 1 × 10^–17^ (V^2^/Hz), which is much lower than the measured signal of the device studied. The details of the growth and circuit design can be found elsewhere^12,15,17^ and in the Supplementary Information.

## Results and discussion

Figure 1b represents the I–V curve of the MTJ device with typical bipolar-type RS characteristics. The device switches from HRS to LRS at the SET voltage of + 1.0 V and from LRS to HRS at the RESET voltage of −1.1 V. Both the HRS and LRS of the device exhibit smooth nonlinear I-V characteristics. To prevent the breakdown of the MTJ, and the dramatically decreasing of MR effect caused by the higher bias voltage, the cycling of I-V curve is kept in the low bias range. Note that the RS channel could be the intermediate states^16^. Our previous result have shown that both HRS and LRS exhibit nonmetallic-like conduction for the temperature dependent measurement of resistance^12^, which suggests that the device undergoes non-metallic filamentary type RS. Figure 1c reveals TMR as a function of magnetic field, a manifestation of the spin-dependent tunneling characteristics, measured in the HRS and LRS. The TMR ratio is defined as:

1 $$TMR\left(\%\right)=\frac{{R}_{AP}-{R}_{P}}{{R}_{P}}\times 100\%,$$

where ${R}_{AP}$ and ${R}_{P}$ are the resistance of the MTJ when the magnetization of the two FM layers is antiparallel and parallel, respectively. The TMR ratio of the HRS and LRS is around 16% and 8%, respectively, at a reading voltage of 50 mV. Furthermore, Fig. 1d displays the TMR ratio taken in accordance with the I-V curve in Fig. 1a. The TMR ratio gradually decreases with increasing bias voltage in the HRS and LRS, showing a typical bias-dependent feature in MTJs^18^. The lower TMR ratio at a higher bias voltage suggests that a decrease in the probability of direct tunneling caused by the presence of ${V}_{O}^{..}$ is responsible for the suppression of the spin-dependent tunneling effect. If the trap level of the ${V}_{O}^{..}$ is close to the Fermi level, it could provide a finite probability for tunneling transport, leading to a smaller TMR in the LRS. In comparison to the previous studies on RS and TMR characteristics in MTJs^19,20^, our results demonstrate that the bias-dependent MR states in the HRS and LRS are strongly correlated with the electrically tunable energy level of the localized ${V}_{O}^{..}$ states in the AlO_x_ barrier.

**Figure 2:**
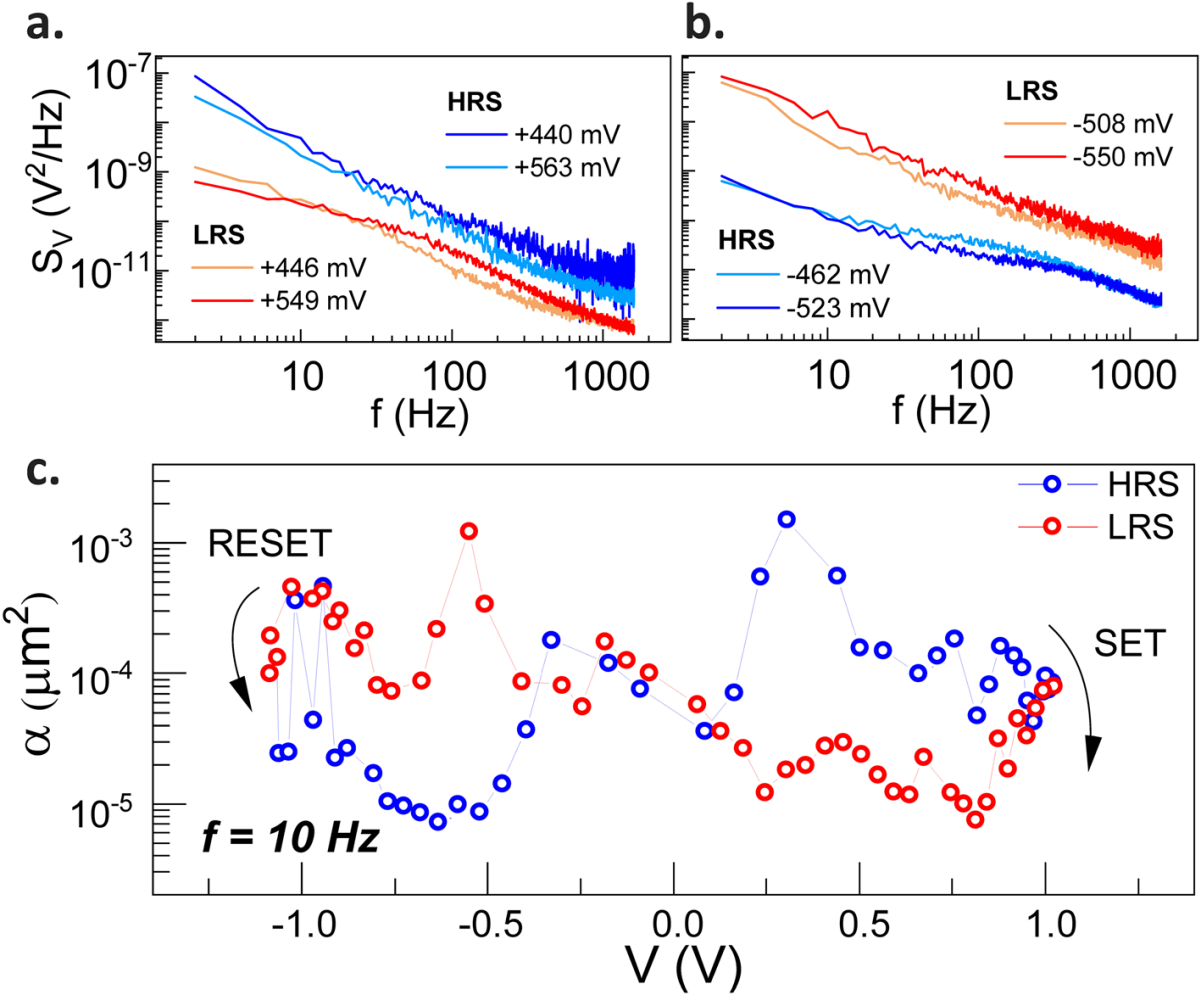
The noise spectrum of HRS and LRS in (**a**) positive and (**b**) negative bias regimes. Note that HRS/LRS exhibits a higher noise level in the positive/negative bias regime, respectively, illustrating a feature of the noise PSD at different bias polarities. (**c**) The bias voltage dependence of the extracted Hooge’s parameter at 10 Hz during a cyclic process of bipolar resistive switching.

To gain more insight into the dynamics of charge transport properties, we conduct LFN measurements to investigate the charge fluctuations of the tunneling effect. The LFN signals were measured from the reading voltage of the designed voltage sweep in the I-V curve, as depicted in Fig. 1a. The voltage noise PSDs of the HRS and LRS taken at ~ $\pm$ 500 mV bias is illustrated in Figs. 2a, b. It appears that a higher noise level of PSD is observed in the positive/negative bias regime for the MTJ studied in HRS/LRS. To facilitate a better understanding of this noise phenomenon, we adopt Hooge’s empirical relation to characterize the low-frequency 1/*f* noise spectrum level by Hooge’s parameter^21^

2 $$\alpha =\frac{Af{S}_{V}}{{V}^{2}},$$

where ${S}_{V}$ is the measured PSD of 1/*f* noise, *V* is the voltage across the junction, and *A* is the cross-sectional area of the junction. Earlier studies have reported that the value of $\alpha$ for different MTJs depends on various physical quantities, such as the resistance-area (*RA*) product, TMR ratio, and bias voltage, but is independent of the bias polarity^21,22^. However, as shown in Fig. 2c, the voltage dependence of the $\alpha$ value for the ultra-thin AlO_x_-based MTJs at a frequency of 10 Hz exhibits an dependence on the bias polarity. Specifically, a broad peak with the highest value of $\alpha$ located around −0.55 V in LRS and 0.3 V in HRS, which is consistent with the feature of the resistive switching, giving rise to large noise level. Thus, even if the bias voltage is not large enough to switch the resistive states, the noise level can still be enhanced due to charge fluctuations or the structure of conductive path. We, therefore, attribute the observed asymmetric noise level to the localized trap-induced dielectric change by resistive switching.

The PSD of RTN can be described by the Lorentzian function^22,23^

3 $${S}_{V (RTN)}=\frac{{S}_{0}}{1+{(f/{f}_{c})}^{2}},$$

where ${S}_{0}$ is the frequency-independent portion of $S_{V(RTN)}$ observed at *f* = 0 Hz and ${f}_{c}$ is the cut-off frequency. Note that ${S}_{0}$ is proportional to the square of RTN magnitude $\Delta V$^24^, and it indicates the intensity of RTN event. For a system with localized states, the total PSD incorporating the contributions from 1/*f* noise and RTN can be expressed as

4 $${S}_{V (total)}=\frac{\alpha {V}^{2}}{Af}+\frac{{S}_{0}}{1+{(f/{f}_{c})}^{2}}.$$

**Figure 3:**
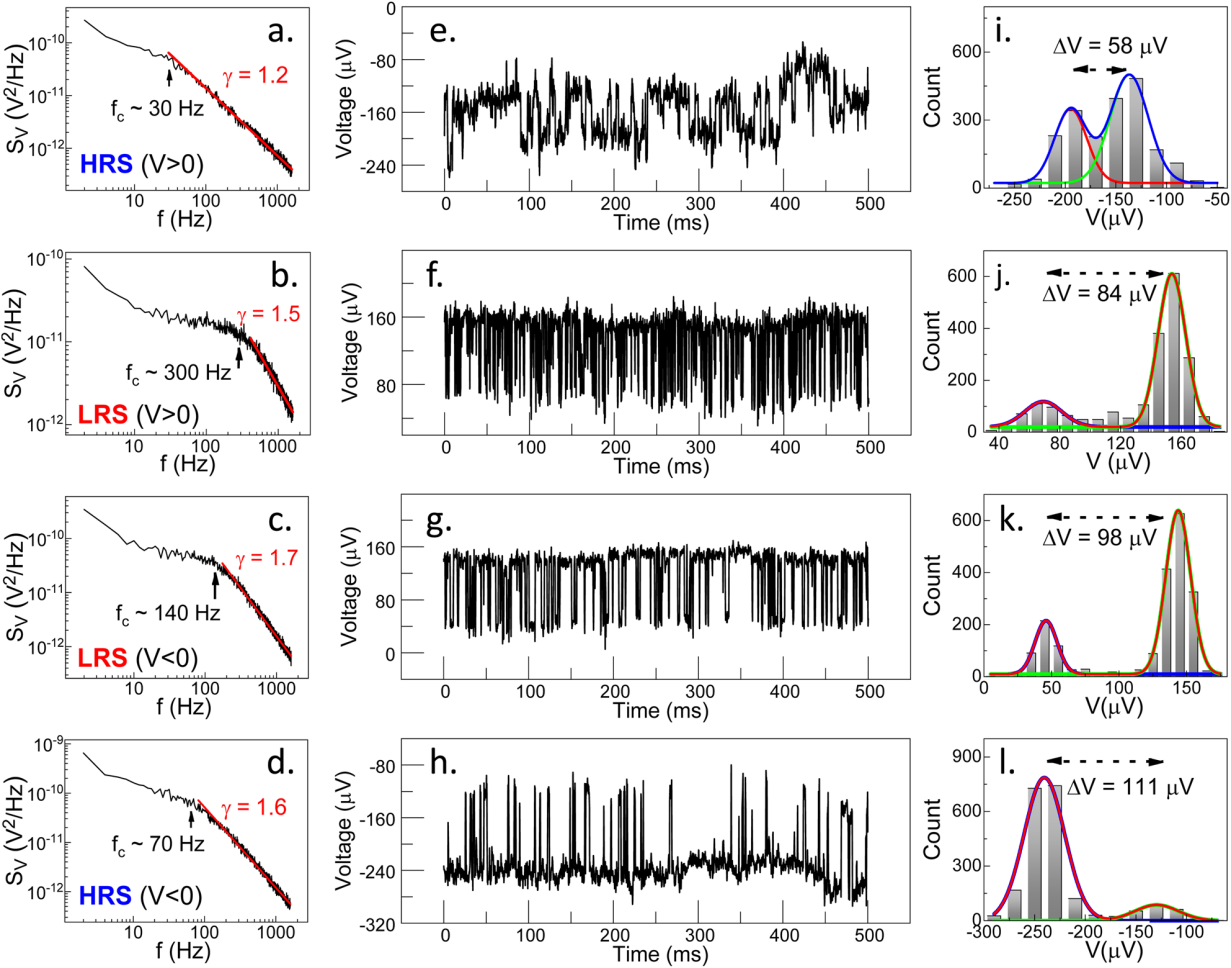
Log-scale noise PSD for (**a–d**) HRS and LRS under different bias polarity with frequency exponent $\gamma$ obtained from fitting a line to the data above the cut-off frequency. (**e–h**) Noise voltage fluctuations in the time domain and (**i–l**) the corresponding histogram with fitting Gaussian peaks for this figure (**a–d**), respectively, in which the $\Delta V$ represents the RTN magnitude. All the signals were measured around a low bias voltage magnitude of 150 mV.

Note that experimental investigations on different systems have reported that the exponent index for the 1/*f* noise falls within the range of 0.9 to 1.4^25^. In our phenomenological approach, we consider ${S}_{V (total)}$ to be proportional to $1/{f}^{\gamma }$. As a result, a PSD with an exponent index larger than 1.4 is predominantly influenced by RTN, whereas an exponent index around 1.0 is primarily governed by 1/*f* noise. To explore the intrinsic noise related to charge transport dynamics, we focus on voltage noise PSD at a low bias magnitude around 150 mV. For positive bias, as shown in Fig. 3a, b, a lower ${f}_{c}$ ~ 30 Hz and $\gamma =1.2$ for the HRS and a higher ${f}_{c}$ ~ 300 Hz and $\gamma =1.5$ for the LRS are extracted from the corresponding noise PSD. For negative bias, Fig. 3c, d show a higher ${f}_{c}$ ~ 140 Hz and $\gamma =1.7$ for the LRS and a lower ${f}_{c}$ ~ 70 Hz and $\gamma =1.6$ for the HRS, which is consistent with the behavior under positive bias that the f_c_ is larger in LRS than that in HRS. In addition, Fig. 3e–h illustrate voltage fluctuations due to RTN with two-level time series for the HRS and LRS, respectively. Overall, the two-level RTN signifies that the electrons undergo capture or emission events by a single localized trap. The histograms of the RTN are well-fitted two Gaussian peaks, as displayed in Fig. 3i–l. Notably, the $\Delta V$ is similar (84/98 μV at ± bias) in LRS with different bias polarities but nonidentical (58/111 μV at ± bias) in that of HRS, indicating that LRS is mainly dominated by a certain local conductive path, such as the ${V}_{O}^{..}$ channels. In contrast, the MTJ in HRS could experience asymmetrical tunneling behavior under different bias polarities. In light of the above, these experimental findings also suggest that the ${V}_{O}^{..}$ could be distributed asymmetrically in the AlO_x_ for the MTJ studied, giving rise to the two different types of RTN and different noise components between HRS and LRS. Further studies, such as temperature/bias dependent analysis of ΔV and relaxation time will be helpful to give quantitatively the location of the ${V}_{O}^{..}$ distribution.

Previous studies have demonstrated that larger magnitude of RTN occurs in the HRS of various oxide-based RS systems^26–28^. It has been also reported that resistance-dependent RTN magnitude depends on the diameter of the conductive filament or the charge trapping by neighbor defect, and that the localized current will be fluctuated by the hopping charge, showing that the RTN magnitude of LRS should be much smaller than in that of HRS^16^. If the LRS is based on the metallic filament formed by the oxygen vacancies, all those oxygen vacancies could be regarded as mediated trap sites, and in turn leads to the superposition of RTN with different relaxation time^27^. As a result, the time series does not reveal clear two-level RTN but tends to display 1/f characteristics. On the contrary, the obvious RTN signals with same order of magnitude in both the HRS and LRS of the present study implies that the charge transport path could be through slightly different distribution of filamentary-like channel governed by the inherently existing ${V}_{O}^{..}$ in the ultra-thin AlO_x_, which is completely different from that in other RS devices for thick oxide layers with conductive filament^26–28^. Moreover, the cut-off frequency ${f}_{c}$ is a measure of the time taken by an electron to transit from the electrode to the first trap site inside the oxide barrier. Consequently, a lower ${f}_{c}$ value corresponds to a longer tunneling length^27^. As discussed earlier, the MTJ studied in the LRS exhibits a typical two-level RTN with a larger ${f}_{c}$ in noise PSD, suggesting that the tunneling length associated with the LRS is shorter than that with the HRS in the ultra-thin AlO_x_ tunneling barrier.

In addition, the interface-type RS has been attributed to a modification of redox reaction at the interface base on the charge trapping and de-trapping at the modified Schottky barrier of the metal-oxide interface in the presence of homogeneous movement of ionic migration^29,30^. Investigations on the interface type of RTN have been conducted in the AlO_x_/PCMO system, showing that opposite magnitudes of RTN in HRS/LRS could be also observed under same bias polarities. This phenomenon is attributed to the different devices with the presence of trap site located at both the AlO_x_/PCMO interface and within the bulk PCMO^31^. On the contrary, the RTN studied in our work shows larger ${f}_{c}$ in LRS under both positive and negative bias, implying that the tunneling electrons undergo smaller tunneling length whether from top or bottom electrode, as compared to that in HRS. And the small difference of $\Delta V$ in LRS could be due to different length/distribution of conductive ${V}_{O}^{..}$ channels in the AlO_x_ under positive/negative bias polarities. In contrast, the $\Delta V$ of HRS under different bias polarity shows larger difference, indicating that the tunneling electrons transit from top and bottom electrode could exhibit different tunneling length and barrier height such that the tunneling current should be extremely affected by the interface and tunneling length. According to the low bias RTN characteristics in HRS and LRS, the possible distribution/location of ${V}_{O}^{..}$ could be recognized. As compared to the previous studies of MTJs with RS^19,20^, our result shows that the TMR effect is observed in both HRS and LRS, indicating that the ${V}_{O}^{..}$ channel in LRS could be noncontinuous to connect top and bottom electrodes. Moreover, the different characteristics of RTN in HRS/LRS represent the conductive path is strongly dependent on the tunneling length and the bias polarity. Notably, combining *f*_c_ with ∆V for the noise characteristics could be a powerful indication to recognize the possible distribution of oxygen vacancies and it's pretty sensitive to different bias polarities. Especially for HRS, in negative bias, the tunneling electrons experience higher ∆V when injected from the top electrode, indicating a higher probability of interaction with trap sites. As for positive bias, tunneling electrons from the bottom electrode experience better tunneling transport, as they preferentially encounter a better barrier before encountering the oxygen vacancy states. According to these results, we suggest that oxygen vacancies may be more localized near the top interface, and that the charge transport in our system could be constructed by the non-metallic ${V}_{O}^{..}$ channel rather than the metallic conductive filament (such as Co and Fe ions) nor the interface type RS.

**Figure 4:**
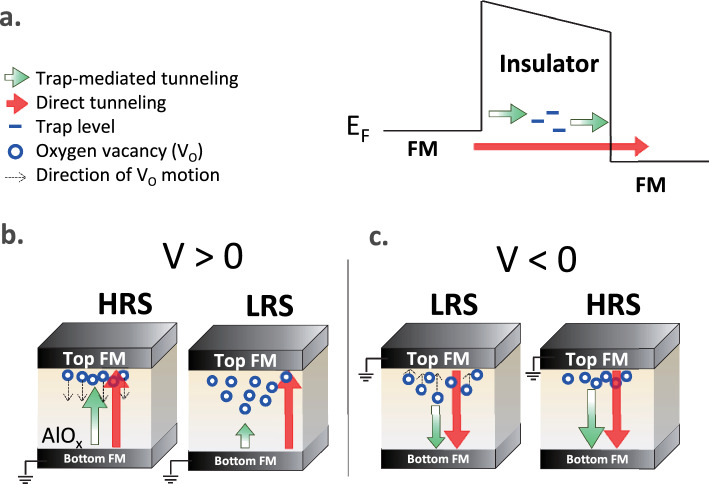
(**a**) The schematic energy-band diagram for the AlO_x_-based MTJs. (**b,c**) The schematic diagram of the charge transport in the HRS and LRS under positive and negative bias, respectively. The red/light green arrow indicates direct tunneling/trap-mediated tunneling process and the blue circle represents the ${V}_{O}^{..}$ trap state. Note that the size of the arrow indicates the probability of a charge transport event, and the dash arrow represents the direction of ${V}_{O}^{..}$ motion in SET/RESET process.

Figure 4 schematically provides a qualitative picture to elaborate on the essential roles played by the inherently existing ${V}_{O}^{..}$ for the charge transport mechanism and two-level RTN signal with a longer/shorter tunneling length observed in the HRS /LRS at a low bias voltage. As the energy level of the trap states associated with the inherent ${V}_{O}^{..}$ lies close to the Fermi level of the FM electrode within a variation of thermal energy *kT*, the probability of the trap-mediated tunneling via the localized traps, in principle, substantially increases, which contributes to the total resistance together with the direct tunneling between the bottom FM and the top FM electrodes, as illustrated in Fig. 4a. For the as-grown MTJ devices, the spatial distribution of ${V}_{O}^{..}$ in the AlO_x_ layer is determined by the two-step oxidation process, yielding a concentration gradient towards the bottom electrode. At a positive low bias, the field- and heat-driven ${V}_{O}^{..}$ migrations are negligibly small. Therefore, the diffusion-driven ${V}_{O}^{..}$ migration is a dominant pathway that leads to the random distribution of ${V}_{O}^{..}$ near the top CoFe/AlO_x_ interface, as shown in Fig. 4b. Consequently, the electrons in the HRS experience a longer tunneling length to encounter the traps near the top interface could be responsible for smaller f_c_, as they are injected from the bottom electrode into the AlO_x_ layer. As the bias voltage increases, the MTJ studied switches from the HRS to the LRS (SET process) after the electroforming process upon which the diffusion- and field-driven ${V}_{O}^{..}$ migrations compete with each other. When the steady-state current is reached, the distribution of ${V}_{O}^{..}$ extends to the middle region of the AlO_x_ layer, which result in larger f_c_ in LRS with different bias polarities, as shown in Fig. 3b, c. When the bias undergoes the negative region, the lower f_c_ implies a slight reduction of ${V}_{O}^{..}$ distribution as schematically illustrated in the left diagram of Fig. 4c. As it is switched from the LRS to the HRS, the f_c_ further reduced than that in LRS due to the RESET process, which reflects the sketch of ${V}_{O}^{..}$ distribution in the right diagram of Fig. 4c. Based on the fact that the TMR effect is a manifestation of the spin-preserved tunneling, the spin polarization should be consistent under the HRS and LRS. The lower TMR in LRS could be due to the spin flip by more localized mediated trap sites with the higher hopping probability, such that it will suppress the TMR. A larger/smaller TMR ratio observed in the HRS/LRS strongly suggests that the direct tunneling/trap-mediated tunneling is the predominant charge transport mechanism for the HRS/LRS, respectively.

## Conclusion

To conclude, we observe LFN-bias voltage loop and bias polarity dependent RTN in the ultra-thin AlO_x_-based MTJs. The bias voltage at which the higher noise level is observed in the loop, associated with the resistive switching-induced significant voltage fluctuations, can be viewed as an indicator of the onset of the RS. Furthermore, polarity dependent RTN shows that charge transport properties, including the tunneling length and the weighting of the direct tunneling/trap-mediated tunneling, are different in HRS and LRS. These findings suggest that the inherent ${V}_{O}^{..}$ are distributed near the top CoFe/AlO_x_ interface as the device is in the HRS. In contrast, the ${V}_{O}^{..}$ could be expanded to the middle region of AlO_x_ in the LRS and result in an effective shorter tunneling length. Notably, the location and distribution of the inherent ${V}_{O}^{..}$ channel can be electrically tuned, which plays an essential role in the charge transport dynamics of the ultra-thin AlO_x_-based MTJs. These results shed light on the complexities of the RS behavior in these devices and pave the way for further exploration and optimization of resistive random-access memory technology and spintronic memristors.

### Supplementary Information

Supplementary Information.

## Data Availability

The data that support the findings of this study are available from the corresponding author upon reasonable request.
