# Supplementary material for: Bias polarity dependent low-frequency noise in ultra-thin AlOx-based magnetic tunnel junctions
Source: Sci Rep. 2024 Jun 13;14:13664. doi: 10.1038/s41598-024-59675-3 (PMC11176387; doi:10.1038/s41598-024-59675-3)
Supplement: Supplementary file 1 — Supplementary Information. [file 41598_2024_59675_MOESM1_ESM.docx]

*Scientific Report*

**Supplementary Materials**

***“Bias polarity dependent low-frequency noise in ultra-thin AlOx-based magnetic tunnel junctions”***

**Author**

Chun-Yen Chen^1^, César Gonzalez-Ruano^2^, Isidoro Martinez^2,3^, Farkhad G. Aliev^2^, Dah-Chin Ling^4^, Yu-Hui Tang^1^ and Jhen-Yong Hong^4,*^

**Affiliation**

^1^ Department of Physics, National Central University, Taoyuan city 320317, Taiwan

^2^Departamento Física Materia Condensada, C03, INC and IFIMAC, Universidad Autónoma de Madrid

^3^Departamento FAIAN, E.T.S.I. Aeronáutica y del Espacio, Universidad Politécnica de Madrid, 28040 Madrid, Spain

^4^Department of Physics, Tamkang University, Tamsui Dist. New Taipei City 251301, Taiwan

**Corresponding author**

* jyhong@gms.tku.edu.tw

**Experimental setup for the noise measurement**

Figure S1 illustrates the experimental configuration for the I-V curve and noise measurement of the MTJ devices. The voltage source was generated using the Keithley 2400 Source Measure Unit (SMU). Cross-spectrum technology was employed to characterize noise behaviors, such as 1/f noise and Random Telegraph Noise (RTN). Therefore, the junction voltage was duplicated into two identical signals for two channels. Both channels were further amplified by homemade pre-amplifiers. The DC voltage of the MTJs is read by the Data Acquisition Board (DT322) from the external branch of one of these two channels. It is important to note that the MTJ sample and INA111Amps are shielded by a metal box, and all instruments are chassis grounded to avoid interference from utility power, such as the utility frequencies of 50 Hz and 60 Hz AC signals.

To separate the "DC voltage" and the "Noise" components from the MTJs, we adopted Low Noise Voltage Preamplifiers (SR560), with a cutoff frequency range selected between 1 Hz to 3 KHz. Finally, the signals from the two channels are sent to a spectrum analyzer (SR780) for the acquisition of time-domain signals and the spectrum of noise. The time-domain signal is measured by one capture without amount averaging, while the noise spectrum is captured by the cross-spectrum of two time-domain signals using the Fast Fourier Transform (FFT) method with 100 times the number of averages.


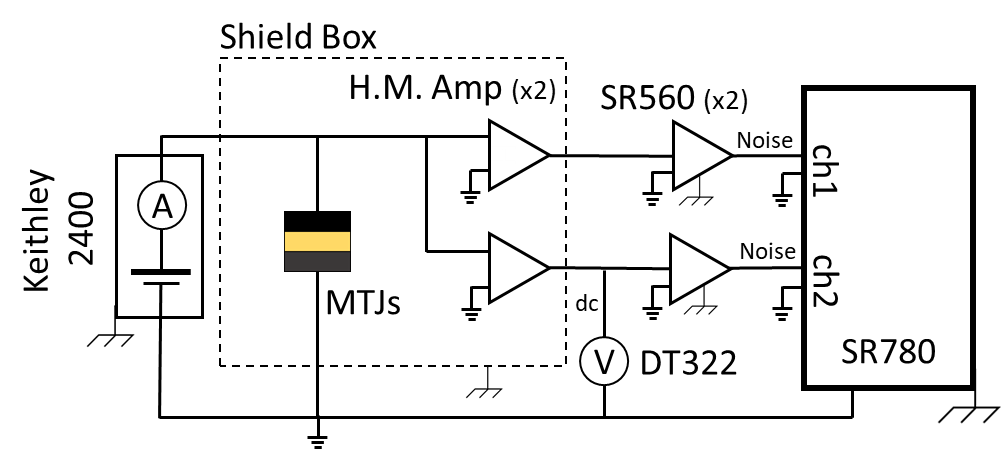


FIG S1. The circuit configuration for the I-V curve and noise measurement.
